# Relationship between soil microbial necromass carbon and community assembly in the forest-grassland ecotone of northern China

**DOI:** 10.3389/fmicb.2026.1802904

**Published:** 2026-07-06

**Authors:** Xuman Ma, Jinhua Liu, Chenghao Li, Yihan Tian, Rui Wang, Xumin Wang, Shuaiming Zhao, Xuehua Xu

**Affiliations:** Department of Ecology, College of Forestry, Hebei Agricultural University, Baoding, China

**Keywords:** forest-grassland ecotone, land-use, microbial community assembly, microbial necromass carbon, soil depth

## Abstract

**Introduction:**

Microbial necromass carbon (MNC) is crucial for the turnover of soil carbon within land ecosystems. Investigating the relationship between microbial residual carbon and microbial community assembly across contrasting land-use patterns provides critical insight into whether land-use modification can regulate microbial community structure to enhance soil carbon sequestration.

**Methods:**

Therefore, this study examined soil MNC and the mechanism of microbial community assembly across the 0-50 cm soil profile of farmlands, grasslands, secondary forests, and planted forests within the forest-grassland ecotone in Northern China.

**Results:**

The results demonstrated significantly higher MNC and accumulation coefficient of grassland in each soil layer compared to the other three land-use types (p < 0.05), with farmland and grassland demonstrating the highest contributions of MNC to soil organic carbon (SOC), accounting for 50.3% and 46.7%, respectively. Increasing soil depth resulted in decreased MNC content in the four land-use types. Fungal necromass carbon (FNC) served as the predominant component within MNC and represented the principal contributor to SOC. Soil bacterial community was regulated by the combined influence of stochastic and deterministic processes across land-use types, whereas fungal community was predominantly driven by stochastic processes. Moreover, a significant association was observed between fungal necromass carbon and the bacterial community assembly process.

**Conclusion:**

The results provide a reference for optimizing land-use strategies within the forest-grassland ecotone of northern China.

## 1 Introduction

Soil microorganisms are integral to the decomposition and stabilization of soil organic carbon (SOC) (Christina et al., 2014). Microorganisms can assimilate plant-derived micromolecular carbon substrates into microbial biomass through *in vivo* turnover pathways. With microbial growth and proliferation, soil carbon ultimately accumulates in the form of microbial necromass. Due to the relatively long turnover time, soil microbial necromass carbon (MNC) can be stabilized through adsorption onto mineral surfaces or physical protection within soil aggregates (Angst et al., 2021; Bai and Cotrufo, 2022). Amino sugars, a typical biomarker, are commonly used to quantitatively characterize the accumulation of MNC and its contribution to soil organic carbon. The high contribution of microbial necromass to SOC suggests that microbial necromass substantially influences soil carbon turnover in terrestrial ecosystems and may improve understanding of SOC stabilization mechanisms (Crowther et al., 2015).

Land-use types change alter plant community structure, which in turn affects microbial groups with distinct life-cycle strategies, ultimately influencing the accumulation of MNC. National-scale studies have indicated that MNC content and its contribution to SOC in farmland are higher than those in grasslands and forests (Wang et al., 2021a). However, some studies have shown the opposite trend (Yang et al., 2026). These discrepancies probably result from the differences in litter chemical composition and decomposition rate among ecosystems (Liang et al., 2017; Trivedi et al., 2016). This explanation aligns with the theoretical framework of microbial life-history strategies. Studies have shown that land-use conversion shifts the microbial community from r-strategy dominance to K-strategy dominance, and this shift is positively correlated with an increase in MNC content (Cai et al., 2025; Lei et al., 2025). Thus, microbial life-history strategies provide a mechanistic basis for understanding land-use effects on MNC accumulation.

Soil depth is also recognized as a key factor regulating MNC content and its contribution to SOC (He et al., 2022). However, there is no consistent conclusion regarding the vertical distribution patterns of MNC. For instance, MNC content increased with soil depth in subtropical forests (Wang et al., 2024). Furthermore, Ni et al. (2020) revealed bacterial necromass carbon (BNC) increased with depth, while FNC remained relatively stable across different soil layers. Nevertheless, opposite distribution trends have been reported in other studies (Sradnick et al., 2014; Yu et al., 2025). Most current researches only focus on the topsoil of a single land-use type, and comparisons of necromass carbon distribution along the complete soil profile under multiple land-use patterns in the same region are lacking.

Microbial community assembly holds a decisive function in the formation and succession of microbial community structure, and is vital for ecosystem functionality. Microbial community assembly reflects the relative balance between deterministic and stochastic processes, which serves as the core mechanism regulating community structure (Zhou and Ning, 2017). Deterministic processes emphasize the shaping effect of environmental filtering on community composition, while stochastic processes focus on the influences of unpredictable factors such as dispersal (Huang et al., 2019). The relative importance of the assembly process may vary under different land use patterns (Chen et al., 2023; Cheng et al., 2021). More importantly, recent studies have begun to reveal the intrinsic relationship between community assembly and necromass carbon accumulation. Zhu et al. (2024) found deterministic assembly processes of bacteria reduce the contribution of MNC, whereas stochastic assembly processes of fungi facilitate necromass carbon accumulation. This finding suggested that the community assembly process may regulate the accumulation and sequestration of necromass carbon by selecting microbial taxa with distinct capacities to produce necromass. However, in such an environmentally sensitive ecotone, the mechanism linking community assembly and necromass carbon remains poorly understood.

The forest-grassland ecotone is an important ecological transitional type distributed in northern China, characterized by vulnerability and instability, which is the combined result of environmental factors and long-term human agricultural and pastoral activities. Due to the impacts of human activities over the years, land use types dominated by secondary forests, plantation forests, grasslands and farmlands have formed in this region. The forest-grassland ecotone in northern Hebei Province is a core area of the northern forest-grassland ecotone, encompassing the above major land use types. Researches on land use types in this region have mainly focused on soil nutrient limitation and microbial community composition (Ma et al., 2025). However, our understanding of soil MNC and its contribution to soil organic carbon, the processes of microbial community assembly, and the relationship between MNC and community assembly under different land use types remains limited. This constrains our understanding of the role of soil MNC in ecosystem carbon turnover and its stability under future land use change scenarios.

In this study, we investigated the relationship between soil microbial necromass carbon and community assembly under different land use types in the forest-grassland ecotone of northern China. The objectives of this study were to (i) analyze the content of soil MNC and its contribution to SOC under different land use types; (ii) reveal the processes of soil microbial community assembly across different land use types; and (iii) elucidate the relationship between soil MNC content and microbial community assembly. We hypothesized that: (i) MNC content and its contribution to SOC vary with land use type and soil depth; (ii) microbial community assembly processes differ among land use types; and (iii) there is a significant association between microbial community assembly processes and MNC accumulation.

## 2 Materials and methods

### 2.1 Research site and soil sampling

The research location is located in Laowopu Township, Weichang County, Chengde City, Hebei Province (N 42°2′7.439″, E 116°59′39.372″). The region has a continental monsoon plateau mountain climate, with an annual average temperature of 5.1 °C and an annual average precipitation of 373 mm. The average altitude is 1,021 m.The region is located at the ecological transition zone between the northern mountains of Hebei Province and the Inner Mongolia grasslands. The research site was originally a secondary forest dominated by *Betula platyphylla*. After forest clearing in the late 1970s, four land-use types were established, including farmland, grassland, *Betula platyphylla* forest, and *Larix principis-rupprechtii* plantation. The farmland follows a maize-sunflower-potato rotation system, while the grassland is of natural origin (Ma et al., 2025). An overview map of the study area and sampling points is shown in Supplementary Figure 1. Detailed information on sampling sites is provided in Supplementary Table 1.

From July to August 2023, the research was conducted on the soil samples collected from four distinct land-use types. Three plots (20 m × 30 m) were established for each land-use type, resulting in a total of 12 plots. Soil samples were collected from five soil profiles at each plot using the five-point sampling method, with samples taken at five depth intervals: 0–10, 10–20, 20–30, 30–40, and 40–50 cm. Five soil samples (one from each profile) from the same layer within a plot were pooled, after removal of plant roots and gravel, and homogenized. A portion of the fresh soil was passed through a 2-mm sieve. and subsequently divided into subsamples for different analyses. One subsample was stored at 4°C or phospholipid fatty acid analysis, while another was flash-frozen using dry ice and preserved at −80 °C for high-throughput sequencing of soil microbial DNA. After air-drying, the remaining soil samples were used for the determination of physicochemical indices and amino sugar content.

### 2.2 Analysis of soil properties

The determination of SOC content was carried out using the potassium dichromate oxidation method under external heating conditions, while TN was extracted through digestion with sulfuric acid, followed by quantification using an Automatic Kjeldahl apparatus (K1305, Sonnen, Shanghai, China). Dissolved organic carbon (DOC) in soil was analyzed with a TOC analyzer (multiN/C 2100S, Analytik Jena, Germany) following extraction with deionized water, while the determination of readily oxidizable organic carbon (ROC) content was carried out through potassium permanganate oxidation, followed by subsequent analysis through a spectrophotometer (TU-1810, Persee, Beijing, China). The particulate organic carbon (POC) content was extracted from soil using sodium hexametaphosphate and quantified through an external heating method using potassium dichromate. Moreover, the chloroform (CHCl_3_) fumigation—K_2_SO4 extraction method was used for the determination of microbial biomass carbon (MBC) content (Bao, 2000).

### 2.3 Determination of microbial phospholipid fatty acid content in soil

Phospholipid fatty acids (PLFAs) were used as specific biomarkers to distinguish different soil microbial groups. Gram-positive bacteria (G^+^) were identified by i14:0, i15:0, a15:0, i16:0, i17:0, and a17:0, while Gram-negative bacteria (G^−^) were characterized by 16:1ω7c, 18:1ω7c, cy17:0, cy19:0, 16:1ω9c, and 17:1ω8c (Wen et al., 2023a), with the sum of Gram-positive and Gram-negative bacteria representing the bacteria (B). Fungi (F) were characterized by 18:2ω6c, 18:1ω9c, 18:2ω6,9, 18:3ω6c, and 18:3ω3c. Arbuscular mycorrhizal fungi (AMF) were identified by 16:1ω5c, while actinomycetes (ACT) were characterized by 10Me16:0, 10Me17:0, and 10Me18:0. Moreover, G^+^/G^−^ and B/F were used to evaluate microbial community structure (Wen et al., 2023a; Zelles, 1997).

### 2.4 Analysis of soil microbial community structure

The microbial genomic DNA was isolated from the soil samples using a DNA extraction kit (DP812, Tiangen, Beijing, China). The primers were designed in accordance with the conserved regions of fungal and bacterial genes. The V3–V4 region of bacterial 16S rRNA was amplified through polymerase chain reaction (PCR) employing primers 338F (5′-ACTCCTACGGGAGGCAGCA-3′) and 806R (5′-GGACTACHVGGGTWTCTAAT-3′) (Caporaso et al., 2011), while the amplification of the ITS1-F region of fungal gene was performed using primers ITS1F (CCTGGTCATTTAGAGGAAGTAA) and ITS2R (GCTGCGTTCTTCATCGATGC) (Buée et al., 2009). The PCR products were purified, quantified, and normalized to generate a high-quality sequencing library for subsequent sequencing analysis. High-throughput sequencing was carried out employing the Illumina NovaSeq 6000 to generate sequencing data. Raw reads were quality-filtered using Trimmomatic v0.33, and primer sequences were removed using Cutadapt v1.9.1 to obtain clean reads. Denoising, paired-end read merging, and chimera removal were performed using the DADA2 plugin in QIIME2 v2020.6, resulting in amplicon sequence variants (ASVs) resolved at 100% sequence similarity. Furthermore, taxonomic annotation of bacterial and fungal ASVs was conducted using the SILVA and UNITE databases, respectively. For downstream analyses, non-target sequences (including chloroplasts, mitochondria, and other non-bacterial sequences in the 16S rRNA dataset, as well as non-fungal sequences in the ITS dataset) were removed. Moreover, ASVs with extremely low abundance (total read counts < 10 across all samples) were excluded to reduce potential sequencing noise. To minimize the biases associated with unequal sequencing depth among samples, ASV tables were rarefied to the minimum sequencing depth across samples before community composition analyses.

### 2.5 Analysis of amino sugar content in soil samples

Soil MNC was measured through gas chromatography-mass spectrometry (Agilent 7890A−5975C, USA). The retention times were recorded for both the sample and the standard, and amino sugar contents were quantified by comparing the peak areas of the sample chromatograms with those of the standards. The inositol was incorporated into each sample before purification and served as an internal standard to enable accurate quantification of amino sugars. All subsequent methodological details are provided in Supplementary Materials (S2). MNC content was calculated using the following formula (Appuhn and Joergensen, 2006):


$$\begin{array}{r} BNC\;=A_{MurA}\times 45\\ \text{FNC }=\left[\frac{{\text{A}}_{\text{GluN}}}{179.17}-2\times \frac{{\text{A}}_{\text{MurA}}}{251.23}\right]\times 179.17\times 9\\ MNC=\left(BNC\right)+\left(FNC\right) \end{array}$$

Here, A_MurA_ is muramic acid content (mg/kg); A_GluN_ represents the glucosamine content (mg/kg); FNC is the fungal necromass carbon; BNC denotes the bacterial necromass carbon; MNC is total MNC in soil; 9 represents the conversion coefficient for the transformation of fungal-derived A_GluN_ into FNC, while 45 is the conversion coefficient for the A_MurA_ transformation into BNC (Appuhn and Joergensen, 2006; Joergensen, 2018).

### 2.6 Soil microbial necromass accumulation coefficient

Microbial necromass accumulation coefficient (NAC) in soil was calculated by using the following formula (Zhang et al., 2021):


$$\begin{array}{l} \text{NAC}=\frac{\text{MNC}}{\text{MBC}} \end{array}$$

### 2.7 Analysis of microbial community assembly

To distinguish the relative roles of deterministic processes (environmental selection) and stochastic processes (dispersal, drift) in community turnover, we employed a null model framework. The phylogenetic β-nearest taxon index (βNTI) was calculated based on phylogenetic distance to assess whether phylogenetic turnover between communities deviates significantly from random expectation. βNTI < −2 indicates homogeneous selection (community phylogenetic structure becomes more similar), βNTI > 2 indicates variable selection (community phylogenetic structure becomes more divergent), and |βNTI| < 2 suggests dominance of stochastic processes (Sun et al., 2025).

For communities with |βNTI| < 2, the Raup-Crick metric based on Bray-Curtis dissimilarity (RCbray) was further used to resolve the specific types of stochastic processes. RCbray < −0.95 indicates that the number of shared species between two communities is higher than expected under the null model, reflecting homogenizing dispersal. RCbray > +0.95 indicates that the number of shared species is lower than expected, reflecting dispersal limitation. And |RCbray| < 0.95 indicates the combined effect of weak selection, dispersal, and ecological drift (that is, non-dominant processes) (Nemergut et al., 2013). The null model expectation was generated through 999 random permutations.

### 2.8 Statistical analysis

We used the linear mixed model (LMM) to evaluate the effects of land-use type and soil layer on amino sugars, MNC, and NAC. Considering the impacts of unbalanced sampling and nested structures, plot was set as a random effect, while land-use type and soil layer were defined as fixed effects. For replicated samples collected along soil profiles, we first calculated the mean value of all profile measurements at the same depth within each plot, and used these averaged values as representative data for each plot-depth combination to satisfy the assumption of statistical independence. The LMMs were fitted using the *lme4* package in R(v.3.6.1) (Bates et al., 2015). The Satterthwaite approximation embedded in the *lmerTest* package was adopted to test the significance of fixed effects (Baey and Kuhn, 2023). *Post-hoc* pairwise comparisons were performed via the emmeans package for significant fixed effects, and the Tukey method was used for *P*-value correction. All statistical analyses were conducted at a significance level of α = 0.05.

Soil microbial community similarity was determined in accordance with the Bray-Curtis (RCbray) distance using the *vegan* package of R(v.3.6.1). The *Picante* package in R(v.3.6.1) was employed to carry out the null model analysis of β–nearest taxon index (βNTI) and the Raup-Crick index based on RCbray, to quantify the relative contributions of deterministic and stochastic processes in community assembly. The *Stats4* and *Hmisc* packages in R were used to implement a neutral model, aiming to determine the regulatory effect of stochastic processes on the microbial community assembly.

The relationships of MNC with biotic and abiotic factors were assessed using a hotmap Furthermore, redundancy analysis was employed to explore the key environmental factors influencing the MNC content within the soil. A random forest model was applied to examine the relationships among βNTI value, environmental factors, and MNC, and to evaluate the relative significance of various environmental factors. Stepwise regression further demonstrated a significant relationship between community assembly and residual carbon content.

## 3 Results

### 3.1 Variations in amino sugars and MNC content in soil

LMM analysis showed that land-use type had a highly significant main effect on all four amino sugars (*p* < 0.001), whereas soil depth alone had no significant main effect on any of them. The interaction was significant for GluN (*p* < 0.001), GalN (*p* = 0.003) and MurA (*p* = 0.042), but not for ManN (*p* = 0.12). These results indicate that land-use is the dominant factor controlling amino sugar concentrations, and that the effect of land-use varies with depth for most amino sugars, except for ManN, where the land-use effect was consistent across depths (see **Supplementary Tables**).

**Figure 1:**
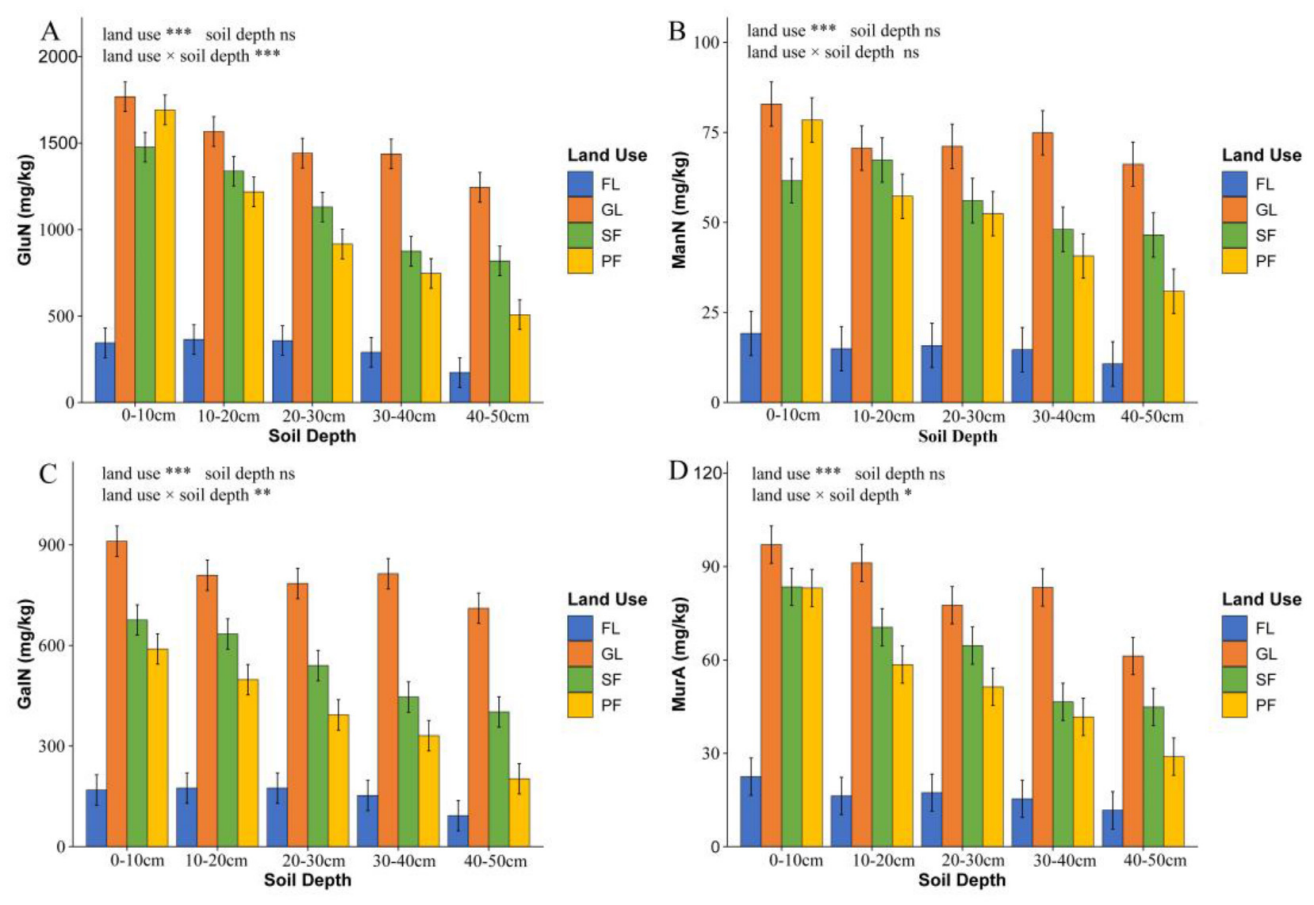
Changes in the amino sugar across various land-use types. **(A)** glucosamine content; **(B)** mannosamine content; **(C)** galactosamine content; **(D)** muramic acid content. FL, farmland; GL, grassland; SF, secondary forest; PF, planted forest. Bars represent mean values ±SE (*n* = 60). ^*^ *p* < 0.05, ^**^p < 0.01, ^***^p < 0.001.

Grasslands generally demonstrated the highest concentration of amino sugar monomer, followed by secondary and plantation forests (Figure 1), with the lowest content observed in the farmland, indicating higher soil microbial biomass and stronger residual carbon accumulation potential within grassland ecosystems. Across all four land-use types, amino sugar content was determined to be decreased with increasing soil depth. In the 0–10 cm layer, GluN and ManN contents were higher in planted forests than in secondary forests, whereas secondary forests showed higher amino sugar contents in the deeper soil layers (Figures 1A,B).

**Figure 2:**
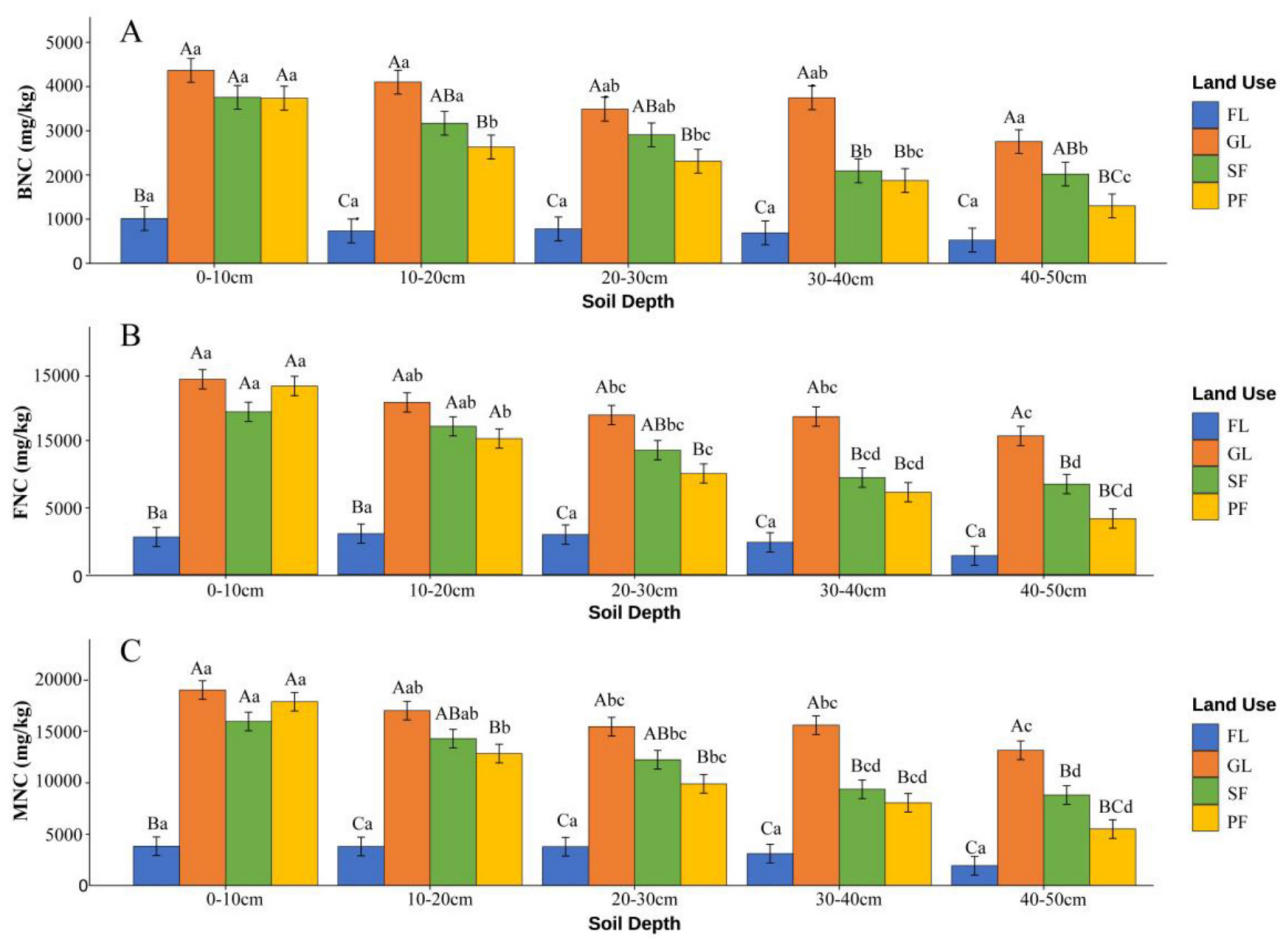
Variations in MNC contents across varying land-use types. **(A)** bacteria necromass carbon; **(B)** fungi necromass carbon; **(C)** microbial necromass carbon. Different uppercase letters indicated significant difference among different land use types within the same soil layer, while different lowercase letters indicated significant difference among different soil layers within the same land use type. Significance was determined using Tukey's post-hoc tests following LMMs (*p* < 0.05).

Grassland demonstrated the highest contents of BNC, FNC, and total MNC among the four land-use types, while the farmland showed the lowest levels. MNC contents in all soil layers of farmland were significantly lower than those in grassland, secondary, and planted forests (*p* < 0.05) (Figure 2C). Throughout the vertical soil profile, the BNC, FNC, and total MNC exhibited a decreasing trend with increasing soil depth under all land-use types. Furthermore, MNC content in the 0–10 cm surface layer remarkably exceeded (*p* < 0.05) compared with the subsoil layer (40–50 cm) across all types. Moreover, the FNC concentration exceeded the BNC content (Figures 2A, B). The results demonstrated significantly higher contribution of fungal necromass to soil microbially-derived organic carbon compared to the bacterial necromass. Soil MNC demonstrated a pronounced vertical stratification.

**Figure 3:**
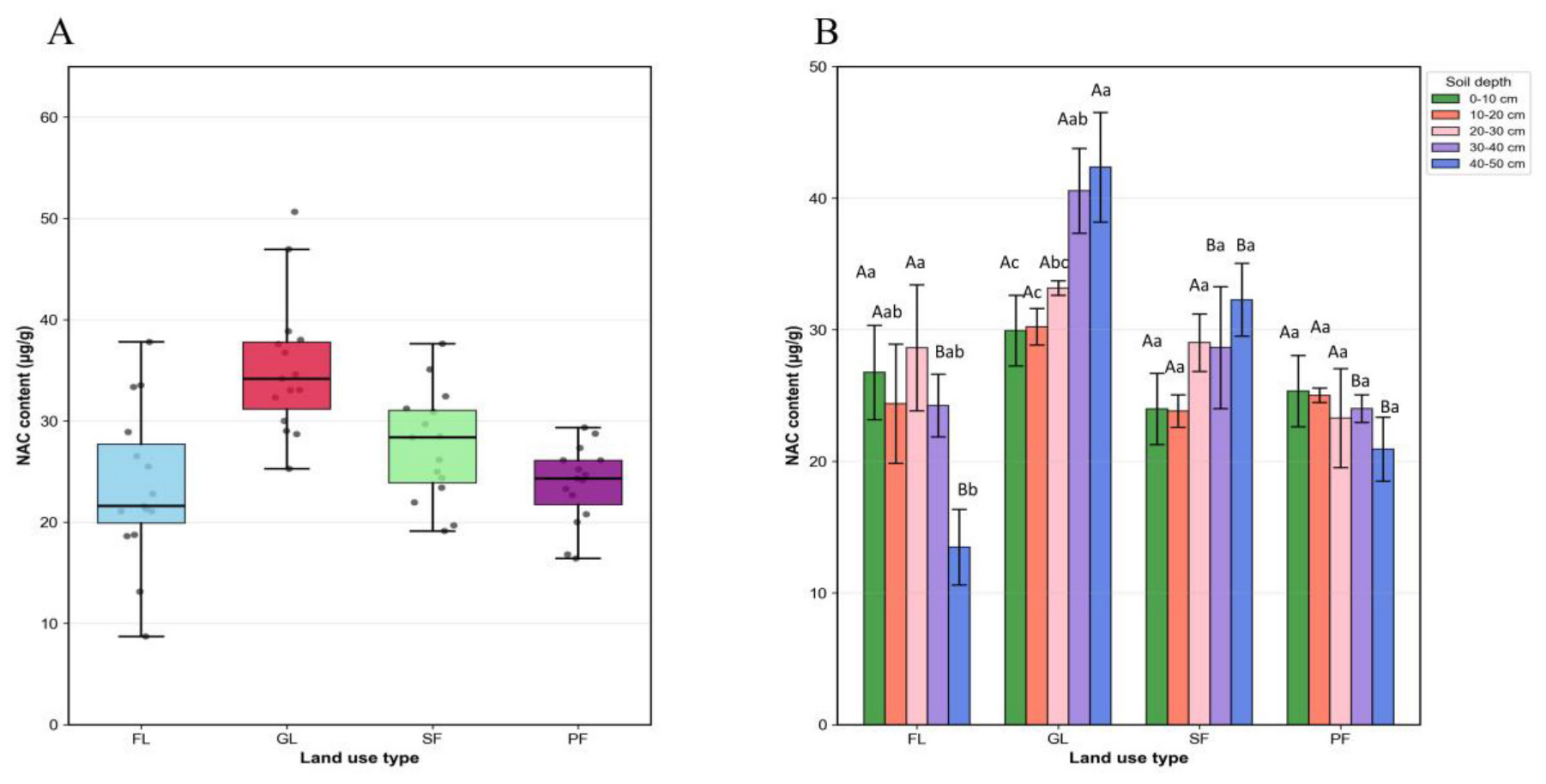
Variations in NAC across different land-use types. **(A)** Boxplot of soil NAC content under different land use types; **(B)** NAC contents in different soil depths across four land use types. LMMs indicated that the main effect of land use patterns is not significant, while the main effect of their interaction is significant (*p* < 0.001).

Among four land-use types, grassland demonstrated the highest MNC accumulation efficiency, while farmland showed the lowest (Figure 3A). Both the grassland and secondary forest demonstrated increased MNC accumulation potential with soil depth, indicating higher carbon sequestration in the deeper layers (Figure 3B). Farmland displayed significantly lower (*p* < 0.05) MNC accumulation within the 40–50 cm layer compared to shallow layers, highlighting the lower carbon sequestration of subsurface soils. Plantation forests showed a unique pattern, characterized by a gradual decline in MNC accumulation efficiency with increasing soil depth.

### 3.2 Contribution of MNC to SOC across different land-use types

**Figure 4:**
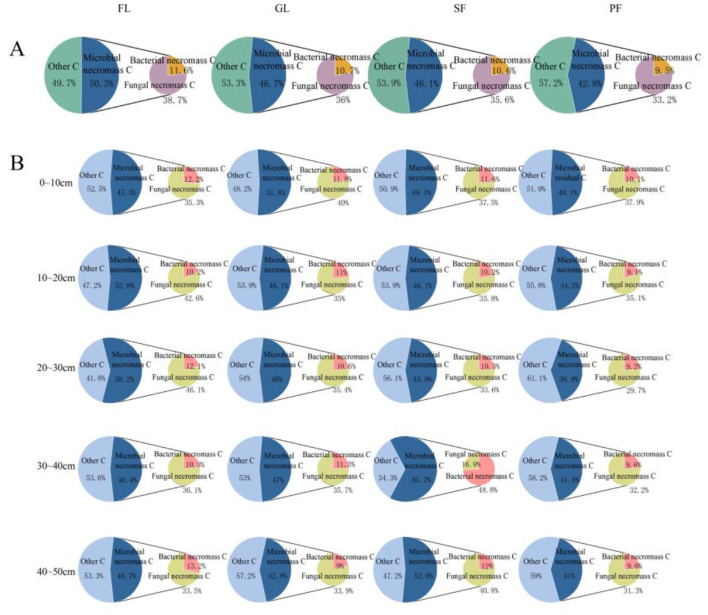
The contribution of MNC to SOC across distinct land-use types **(A)** and soil layers **(B)**.

MNC to SOC contribution ratios in farmland and grassland were determined to be 50.3% and 46.7%, respectively, surpassing the secondary forest and plantation (Figure 4A). This demonstrated higher SOC accumulation potential of farmland and grassland compared with the secondary and plantation forests, where SOC formation was mainly driven by microbial internal turnover processes (intra-organism turnover). Across all four land-use types, FNC constituted the predominant fraction of total MNC, indicating the fungi as the primary source of necromass carbon.

Across the four land-use patterns, the contribution of MNC to SOC varied with soil depth (Figure 4B), with farmland demonstrating an initially increased MNC content, followed by a subsequent decrease with increasing depth, while secondary forest demonstrated the reverse trend. Grassland and plantation revealed a gradual decline in MNC contribution with increasing depth. FNC dominated the MNC contributions across all soil layers, except the 30–40 cm layer in secondary forest, demonstrating predominance of FNC.

### 3.3 Assembly of soil microbial communities

**Figure 5:**
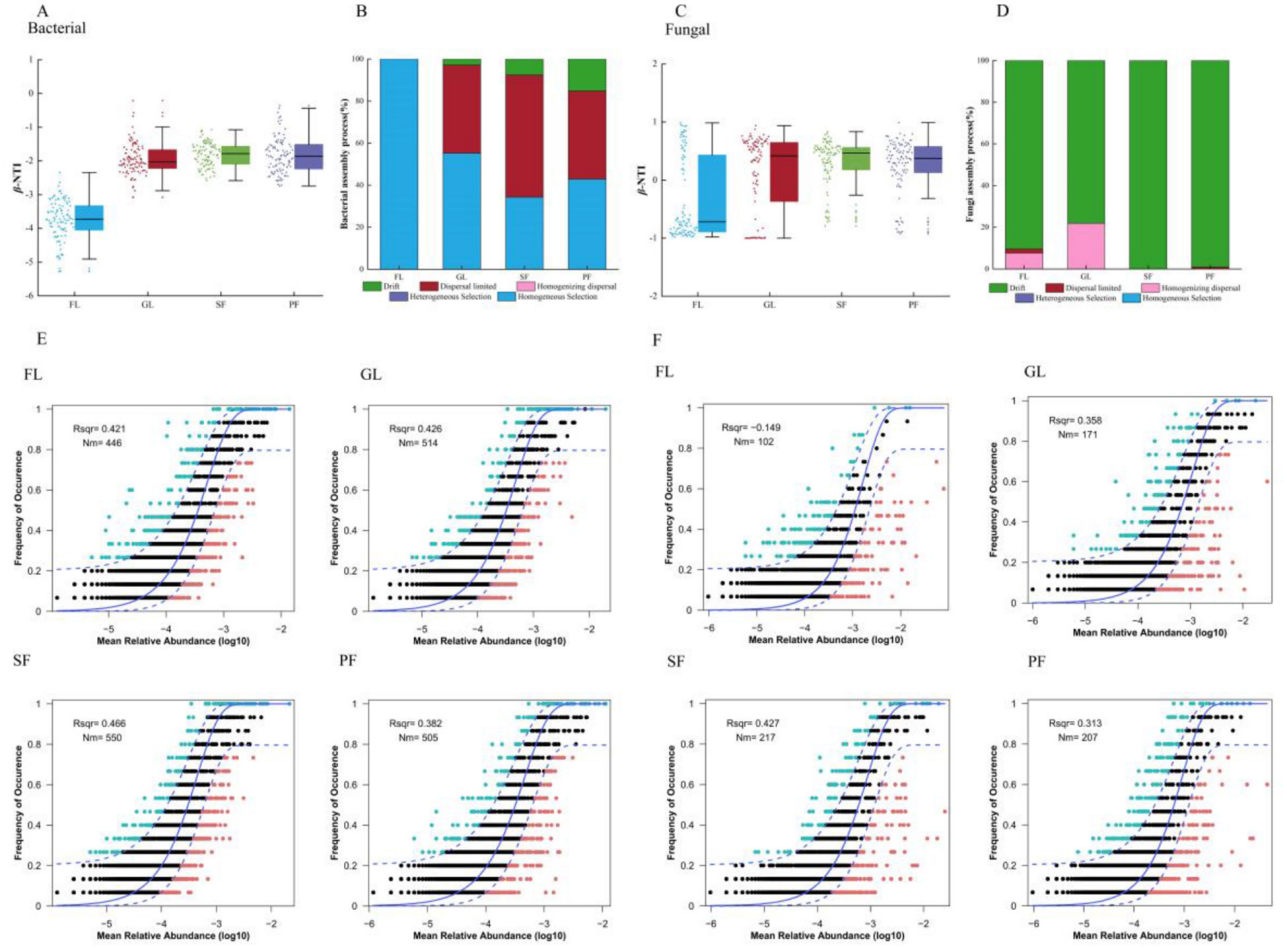
The process of microbial community assembly across distinct land-use types. **(A, C)** β nearest taxon index(βNTI)boxplot of bacterial and fungi communities; **(B, D)** Proportion of ecological processes in bacterial and fungal community assembly; **(E, F)** Neutral model fitting of bacterial and fungal communities. βNTI quantifies phylogenetic turnover between communities: |βNTI| > 2 indicates deterministic selection (homogeneous or variable selection) dominates community assembly, whereas |βNTI| < 2 suggests that stochastic processes, including dispersal limitation, homogenizing dispersal, and ecological drift, are the primary drivers of community assembly. When |βNTI| < 2, RCbray was used to further differentiate the stochastic processes: RCbray > 0.95 indicates dispersal limitation, RCbray < −0.95 signifies homogeneous dispersal, and −0.95 ≤ RCbray ≤ 0.95 indicates ecological drift. The solid line denotes the predicted occurrence frequency, while the dashed lines represent 95% confidence intervals. Neutral model plots show species occurrence frequency against mean relative abundance (log_10_). Nm (metacommunity size × migration rate) represents the migration capacity of taxa (higher Nm indicates stronger dispersal), and Rsqr is the goodness-of-fit of the neutral model.

βNTI analysis indicated that stochastic and deterministic processes regulate the soil bacterial community assembly across the four land-use types, while fungal assembly is primarily stochastic. Among four land-use types, farmland demonstrated the strongest influence of deterministic processes on bacterial communities, with homogeneous selection being particularly prominent (Figure 5A). This suggests that agricultural soils are more responsive to environmental variability and spatial heterogeneity than other land-use systems. Dispersal limitation in the secondary forest revealed a greater contribution compared to the grassland, plantation, and farmland (Figure 5B). Across the four land-use types, the contribution of ecological drift followed the order: plantation > secondary forest > grassland > farmland. All fungal communities demonstrated |βNTI| < 2 (Figure 5C), indicating dominance of stochastic processes across all land-use types. In the secondary forest and plantation, the fungal communities were typically modulated by the drift process. In the farmland, the contributions of homogeneous dispersal, dispersal limitation, and drift to fungal community assembly were determined to be 7.62%, 1.9%, and 90.48%, respectively. In the grassland, the homogeneous dispersal and drift contributed 21.9% and 78.1%, respectively, to fungal community assembly (Figure 5D).

The neutral community model, as a theoretical framework, was used to quantify the influence of stochastic processes on microbial community assembly across different land-use systems. Secondary forests demonstrated the best model fit for the bacterial community (*R*^2^ = 0.466), while plantations showed the weakest (*R*^2^ = 0.382) (Figure 5E), indicating the relatively stronger influence of stochastic processes on bacterial community assembly in secondary forests compared with other land-use types. Furthermore, the fungal community in secondary forest showed relatively higher goodness of fit (*R*^2^ = 0.427) compared to those under other land-use types, indicating a stronger influence of stochastic processes on fungal community assembly (Figure 5F). The secondary forest demonstrated higher migration and dispersal (Nm) of bacterial as well as fungal communities, compared with other land-use types, suggesting more homogeneous species distribution and enhanced dispersal capability within microbial communities in secondary forests. Comparative analysis revealed that the farmland soil harbored both bacterial and fungal communities with the lowest overall dispersal capacity among the studied land-use types.

### 3.4 Factors affecting the MNC content in soil

**Figure 6:**
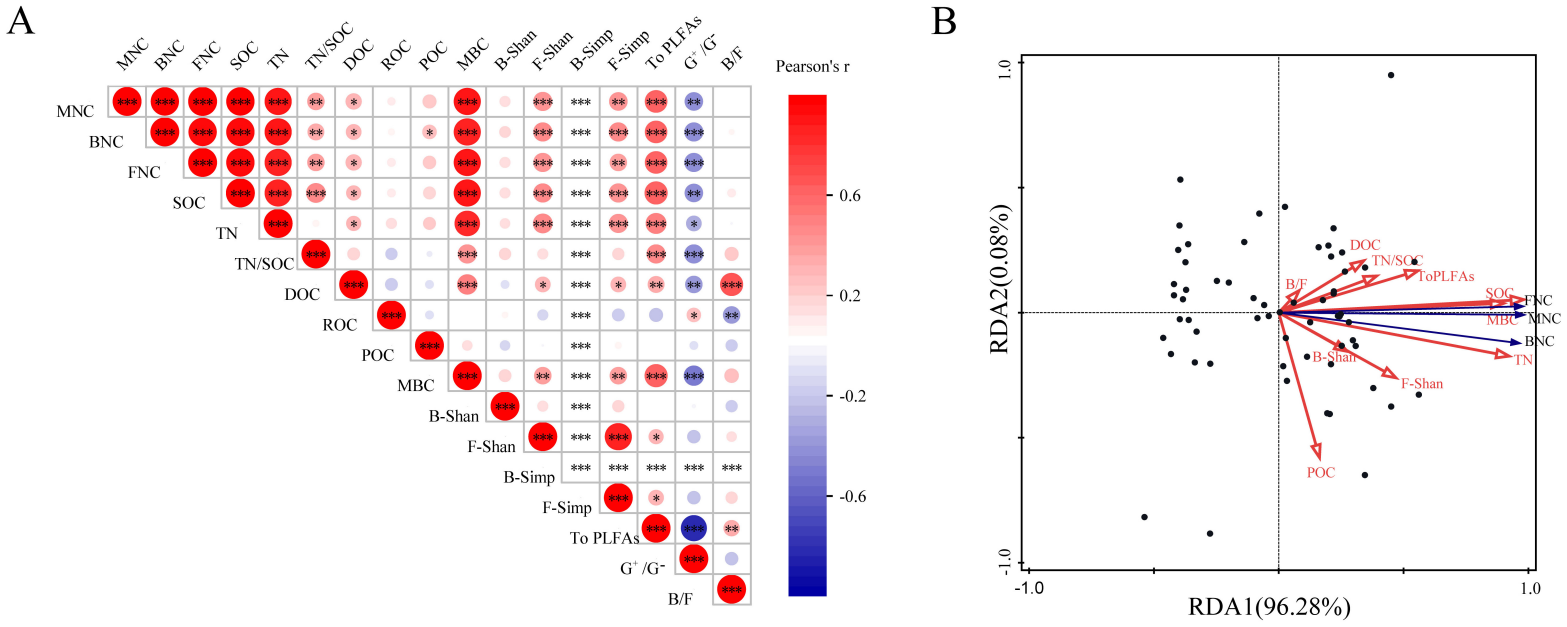
Correlation heatmap **(A)** and redundancy analysis **(B)** of MNC and environmental factors. MNC, total microbial necromass carbon; BNC, bacterial necromass carbon; FNC, fungal necromass carbon; SOC, soil organic carbon; TN, total nitrogen; DOC, dissolved organic carbon; ROC, readily oxidizable organic carbon; POC, particulate organic carbon; MBC, microbial biomass carbon; B-Shan, bacterial Shannon index; F-Shan, fungal Shannon index; G+/G–, Gram-positive/ Gram-negative ratio; B-Simp, bacterial Simpson index; F-Simp, fungal Simpson index; To PLFAs, total phospholipid fatty acids; B/F, bacteria/fungi ratio. ^*^ *p* < 0.05, ^**^p < 0.01, ^***^p < 0.001.

The correlation analysis of soil environmental factors under the four land-use types demonstrated significant positive correlations among SOC, TN, MNC, fungal Shannon index, and fungal Simpson index (*p* < 0.01) (Figure 6A). Furthermore, significant positive correlations of total PLFAs were observed with SOC, TN, TN/SOC ratio, MBC, and bacteria/fungi (*p* < 0.01). G^+^/G^−^ showed significant negative correlations with SOC, TN/SOC, MBC, and total PLFAs (*p* < 0.01). A redundancy analysis of the environmental factors and SOC components (Figure 6B) revealed that the first two axes collectively explained 96.36% of the total variation, with the first axis (96.28%) accounting for the majority of the relationships between MNC and environmental variables, compared to the secondary axis (0.08%). Based on their explanatory power, SOC, TN, and MBC (*p* < 0.01) were identified as the primary environmental determinants influencing MNC content.

### 3.5 The relationship between MNC content and microbial community assembly

**Figure 7:**
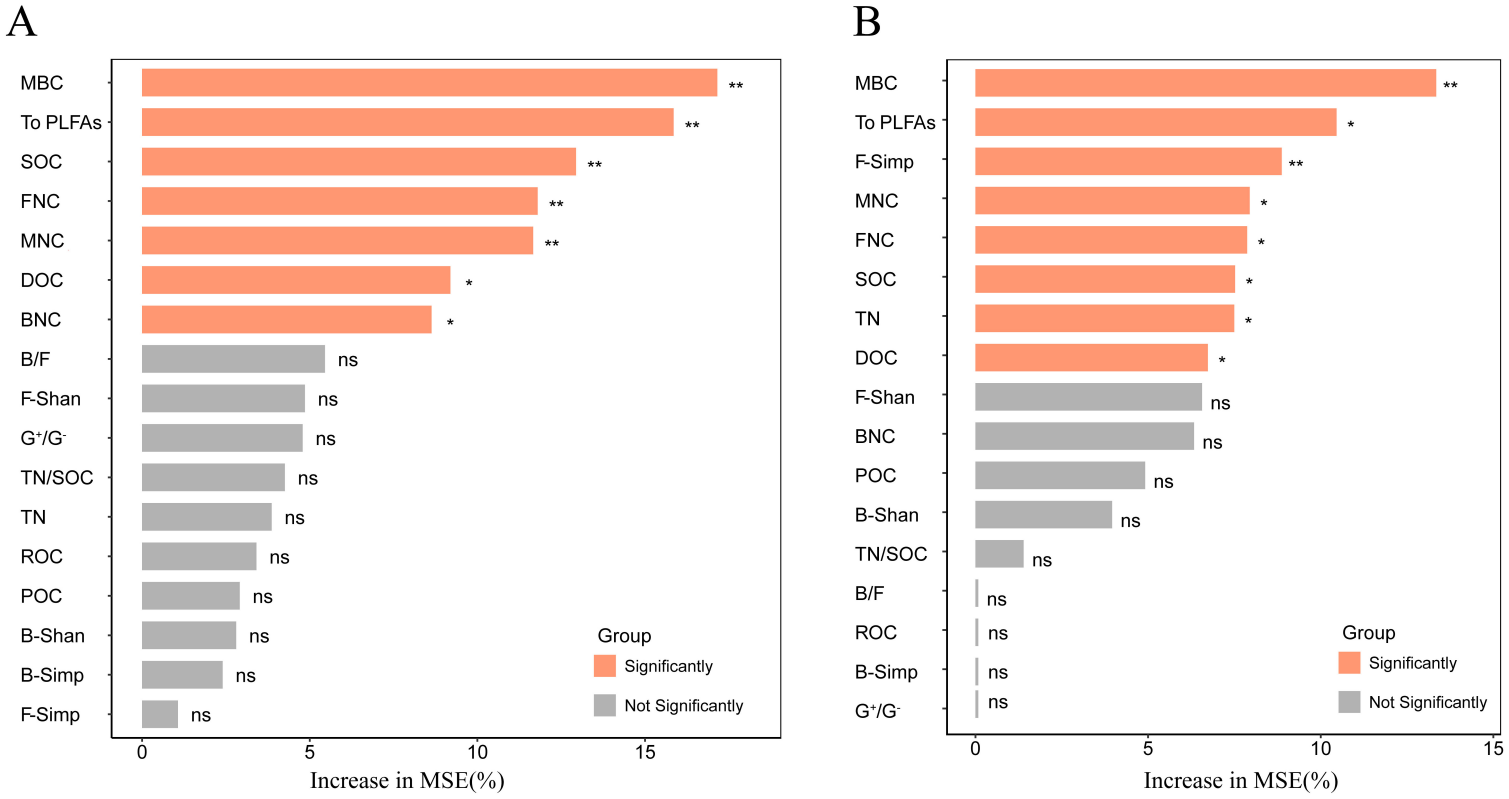
Random forest analysis of bacterial **(A)** and fungal **(B)** community assembly with environmental factors. Increase in MSE (%) represents the contribution of each predictor to model accuracy; higher values indicate stronger association with the response variable. Orange bars represent significant variables (*p* < 0.05, with ^*^ *p* < 0.05, ^**^ *p* < 0.01), while gray bars denote non-significant variables (ns, *p* ≥ 0.05).

Random forest analysis (RFA) demonstrated MBC, FNC, SOC, MNC, and DOC as the variables most strongly associated with bacterial community assembly (Figure 7A). For fungal community assembly, MBC and fungal Simpson index showed the strongest statistical associations (Figure 7B). Stepwise regression showed that bacterial βNTI covaried positively with FNC (*p* < 0.001), and fungal βNTI covaried negatively with MNC (*p* = 0.002) (Table 1).

**Table 1:** Stepwise regression with col-linearity statistics (tolerance and VIF).

Necromass carbon categories	Community assembly types	B	Beta	*t*	*p*	VIF
MNC	B-βNTI	6660.940	0.551	5.574	<0.001	1.016
	F-βNTI	−1708.070	−0.321	−3.241	0.002	1.016
FNC	B-βNTI	5194.017	0.554	5.542	<0.001	0.984
	F-βNTI	−1247.145	−0.302	−3.017	0.004	0.984
BNC	B-βNTI	1466.927	0.524	5.364	<0.001	0.984
	F-βNTI	−460.928	−0.374	−3.822	<0.001	0.984

B, unstandardized coefficient; Beta, standardized coefficient; VIF, variance inflation factor.

## 4 Discussion

### 4.1 MNC content and its contribution to SOC in soils across distinct land-use types

The results demonstrated higher FNC content and its contribution to SOC than those of BNC, consistent with the previous (Ding et al., 2020; Li et al., 2020; Wang et al., 2021a). This result is mainly attributed to the higher living biomass of fungi, the degradation resistance of fungal cell walls, and their competitive advantage in utilizing recalcitrant substrates (Bahram et al., 2018; He et al., 2020). Notably, BNC contribution exceeded that of FNC in the 30–40 cm soil layer of the secondary forest, which was distinctly different from the patterns observed in other soil layers. This may be caused by the restricted fungal growth in this soil horizon of secondary forests, such as reduced aeration and altered forms of available nutrients (Yu et al., 2025). In contrast, bacterial taxa with higher metabolic flexibility gained a competitive predominance at this depth, and their necromass became the primary source of MNC (Schimel et al., 2007).

Land-use type stands emerge as a significant regulating factor of MNC content and its contribution to SOC, as reported by Angst et al. (2021). Compared to the other three land-use types, grasslands showed the highest MNC content and accumulation, consistent with the results of a previous study (Ma et al., 2021). The observed phenomenon was associated with the higher underground biomass of grasslands compared to farmlands, which in turn provides abundant carbon sources for microorganisms. Following microbial decomposition and utilization, a fraction of organic carbon is retained in soils as microbial necromass, which represents an important component of SOC (Prommer et al., 2020). Furthermore, litter-derived carbon from grasslands and farmlands contributes the most to soil organic carbon due to its rapid turnover through microbial decomposition in the subsurface (Liang et al., 2019), thus promoting MNC accumulation (Wang et al., 2021b). Compared with the other lands, farmland exhibited the lowest MNC content and the lowest NAC. This may be attributed to the disruption of the soil micro-food web caused by disturbances such as cultivation. Such disturbances may reduce the predation pressure on bacteria by protozoa, alter microbial community turnover and carbon release pathways, thereby affecting the stability of residual carbon (Asiloglu et al., 2021). Conversely, appropriate agricultural management may mitigate such negative effects. For example, studies on the response of MNC to agricultural management practices have shown that selecting appropriate agricultural management measures based on local conditions can improve soil MNC accumulation (Zhou et al., 2023).

MNC accumulation in soil at different soil depths is governed by varying mechanisms. This study observed the decreased MNC content with the soil depth, consistent with the finding of a previous study on the MNC in various soil layers in the Qinghai-Tibet Plateau (He et al., 2022), likely due to the varying primary drivers of MNC across different soil layers. For example, the surface and deep soils are differently influenced by plant carbon input and mineral protection. However, various studies reported an increased MNC content with soil depth (Ni et al., 2020). The FNC content generally remained consistent throughout the soil profile in forest soils, which may be attributed to multiple factors, such as the decreased plant input and biomass. The MNC contributions to SOC varied across land-use types, increasing and then decreasing with soil depth in farmland, while exhibiting an initial decline followed by a subsequent increase in the secondary forest. Grassland and plantation showed a decreasing trend of MNC contribution as soil depth deepened. This may be attributed to the relatively shallow root systems of crops and grasses, which are unable to effectively retain nutrients in deeper soil layers. The subsoil of secondary forest demonstrated a relatively high MNC accumulation capacity, while subsoils in plantation, grassland, and farmland displayed a weaker capacity, likely due to varying soil properties across different land-use patterns. Wen et al. (2023b) investigated depth-dependent variations in MNC content and reported that MNC accumulation in deep soil layers was controlled by climatic factors, while in topsoil it was co-regulated by soil properties and climate. Therefore, soil properties showed variations in this study under different land-use patterns, affecting soil MNC accumulation.

MNC content in the soil is co-regulated by biotic and abiotic factors (Cao et al., 2023), further highlighting the important roles of MBC as a biotic factor, and SOC and TN as abiotic factors, in regulating soil MNC content. The significant positive correlation between MBC and MNC indicated that the input of plant biomass increased soil MBC content, facilitating MNC formation (Wilpiszeski et al., 2019). Studies have shown that increasing nitrogen content can directly increase microbial biomass growth and indirectly promote MNC accumulation (Wang et al., 2021a; Wu et al., 2025). Moreover, MNC have been reported as a primary constituent of SOC (Miltner et al., 2012), highlighting a close relationship between them.

### 4.2 Microbial community assembly across distinct land-use types

In this research, bacterial community assembly was influenced by both deterministic and stochastic processes, whereas fungal community assembly was primarily governed by stochastic processes, consistent with the results of a previous study (Liu et al., 2024). Importantly, the relative contributions of these processes varied substantially with land-use type (Chen et al., 2023; Kraemer et al., 2020). Our research findings indicate that deterministic processes significantly contribute to bacterial community assembly in agricultural soils, with homogeneous selection being the most important contributor. This may be associated with the strong and consistent selective pressure generated by long-term, homogeneous anthropogenic management in agricultural systems (fertilization, tillage, and monocropping), which could weaken the role of stochastic processes. Similar phenomena have been reported in intensive agricultural systems (Jiao et al., 2020; Shi et al., 2018). In secondary forests, the contribution of dispersal limitation to bacterial assembly was higher than in grasslands and farmlands. Additionally, the neutral model showed the best fit in secondary forests (*R*^2^ = 0.466), indicating the potential importance of dispersal limitation and ecological drift. Homogeneous selection was more pronounced in grassland and plantations. This may be attributed to the stable vegetation input and homogeneous soil microhabitats in grassland and plantation ecosystems, providing a strong environmental screening effect (Huang et al., 2025).

Fungal communities under all land use patterns demonstrated |βNTI| < 2, indicating the significant influence of stochastic processes. Fungal community assembly in secondary forest was predominantly governed by ecological drift, while in farmland, grassland, and plantation, it was co-regulated by both drift and diffusion processes, consistent with the result of the neutral model. A possible reason was that fungal communities may harbor many broadly tolerant species or possess high functional redundancy, which weakens competitive exclusion and environmental filtering and thus favors stochastic processes. Meanwhile, fungal spores generally have long-distance dispersal potential, leading to weak dispersal limitation at the study scale (Golan et al., 2023). In addition, the broader substrate utilization breadth of fungi, including the ability to degrade complex carbon sources, may make them less sensitive to subtle changes in soil properties and thereby weaken the role of deterministic selection (Beidler et al., 2020). This pattern was most pronounced in secondary forests (*R*^2^ = 0.427), where higher habitat heterogeneity further amplified stochastic processes. This result provides a possible background explanation for the statistical association between FNC and bacterial community assembly, but the underlying mechanisms still require further verification.

Indices such as βNTI and RCbray used in this study infer community assembly processes based on phylogenetic and compositional differences. However, their interpretations depend on several key assumptions, such as the accuracy of phylogenetic relationships and the validity of null model construction (Gundersen and Vadstein, 2024). Furthermore, the choice of null models and sample size requirements may also influence the inferred outcomes of community assembly processes (Miller et al., 2017; Smith and de los Reyes, 2024). The neutral community model is based on the assumption of ecological equivalence among species in terms of dispersal capacity and fitness. Therefore, when interpreting the community assembly results of this study, the above-mentioned methodological limitations need to be fully considered. The assembly processes reported in this study should be interpreted as relative contributions operating at a specific spatial scale. Future studies are necessary to incorporate long-term *in situ* experiments and functional gene analyses to establish a more robust causal chain.

### 4.3 Relationship between MNC content and microbial community assembly in soil

This study found a positive correlation between FNC and bacterial community assembly. One possible explanation is that FNC, as a carbon source, favors copiotrophic bacteria, which exhibit high efficiency in utilizing labile necromass carbon. In addition, these fast-growing bacteria are more susceptible to predation by protozoa and other predators, leading to increased mortality and accelerated community turnover (Ma et al., 2023). The combined effect of predation pressure (top-down) and resource availability (bottom-up) may strongly correlate with a deterministic enhancement in bacterial community construction. Therefore, the statistical correlation between FNC content and bacterial community assembly likely reflects the coupled effect of resource selection and predation regulation (Asiloglu et al., 2021; Liu et al., 2024).

This study findings indicated that the establishment of fungal communities is closely correlated with fungal diversity and richness. Higher richness may reflect a larger regional species pool available for ecological selection, improving community adaptability to environmental variability. For instance, Jiao et al. (2021) reported the gradual decrease in the role of stochastic processes with increasing fungal richness, indicating enhanced deterministic processes such as environmental filtering. Furthermore, fungal dispersal ability significantly influences the community structure. Fungi with weaker dispersal limitation can facilitate the colonization of diverse habitats, promoting the community diversity (Fine and Kembel, 2011). Limited dispersal can prevent the excessive expansion of dominant species, thereby providing niche space for the persistence of rare species, and ultimately promoting the maintenance of fungal community diversity.

This study reveals the strong relationship between bacterial community assembly and microbial necromass composition, especially FNC, whereas fungal community assembly is closely related to diversity and richness. However, the experimental design of this study can neither establish a causal relationship between the two nor rule out a common causal pathway (where both respond to the same environmental factors). Future research should integrate multi-omics approaches, multi-trophic ecological network analyses (protists and viruses), and stable carbon and nitrogen isotope tracing techniques to further elucidate the allocation and transformation of microbial necromass carbon, as well as the bidirectional (or co-regulatory) mechanisms between microbial necromass carbon and community assembly, providing a more comprehensive understanding of subsurface carbon cycling processes.

## 5 Conclusion

In this study, the variations in MNC content and community assembly, along with their interrelationships, were examined across four different land-use types in the forest-grassland ecotone of Northern China. Grassland demonstrated the highest concentrations of BNC, FNC, and total necromass carbon, while farmland showed the lowest. Across all land-use types, MNC content demonstrated a decreasing trend with increasing soil depth, with varying contributions to SOC across different soil layers. The null model indicated that stochastic and deterministic processes collectively governed soil bacterial community assembly across all four land-use types. However, stochastic processes exerted a dominant influence overall. Furthermore, the neutral model verified the close relationship between the microbial communities and stochastic processes in the secondary forest. SOC, TN, and MBC significantly affected the MNC content in soil, while FNC content showed significant correlation with bacterial community assembly. This study advances the understanding of soil microbial necromass carbon and community assembly processes across different land use types, and provides a theoretical foundation for optimizing land-use structure in this region.

## Data Availability

The 16S rRNA and ITS data were deposited to the National Center for Biotechnology Information (NCBI) under the project accession number PRJNA1354515 and PRJNA1453694. Other relevant data is publicly available on Mendeley Data: https://data.mendeley.com/datasets/yn8xfxp4p7/1.
