# Supplementary material for: Relationship between soil microbial necromass carbon and community assembly in the forest-grassland ecotone of northern China
Source: Front Microbiol. 2026 Jul 6;17:1802904. doi: 10.3389/fmicb.2026.1802904 (PMC13381471; doi:10.3389/fmicb.2026.1802904)
Supplement: Supplementary file 1 [file Data_Sheet_1.docx]

Supplementary Material


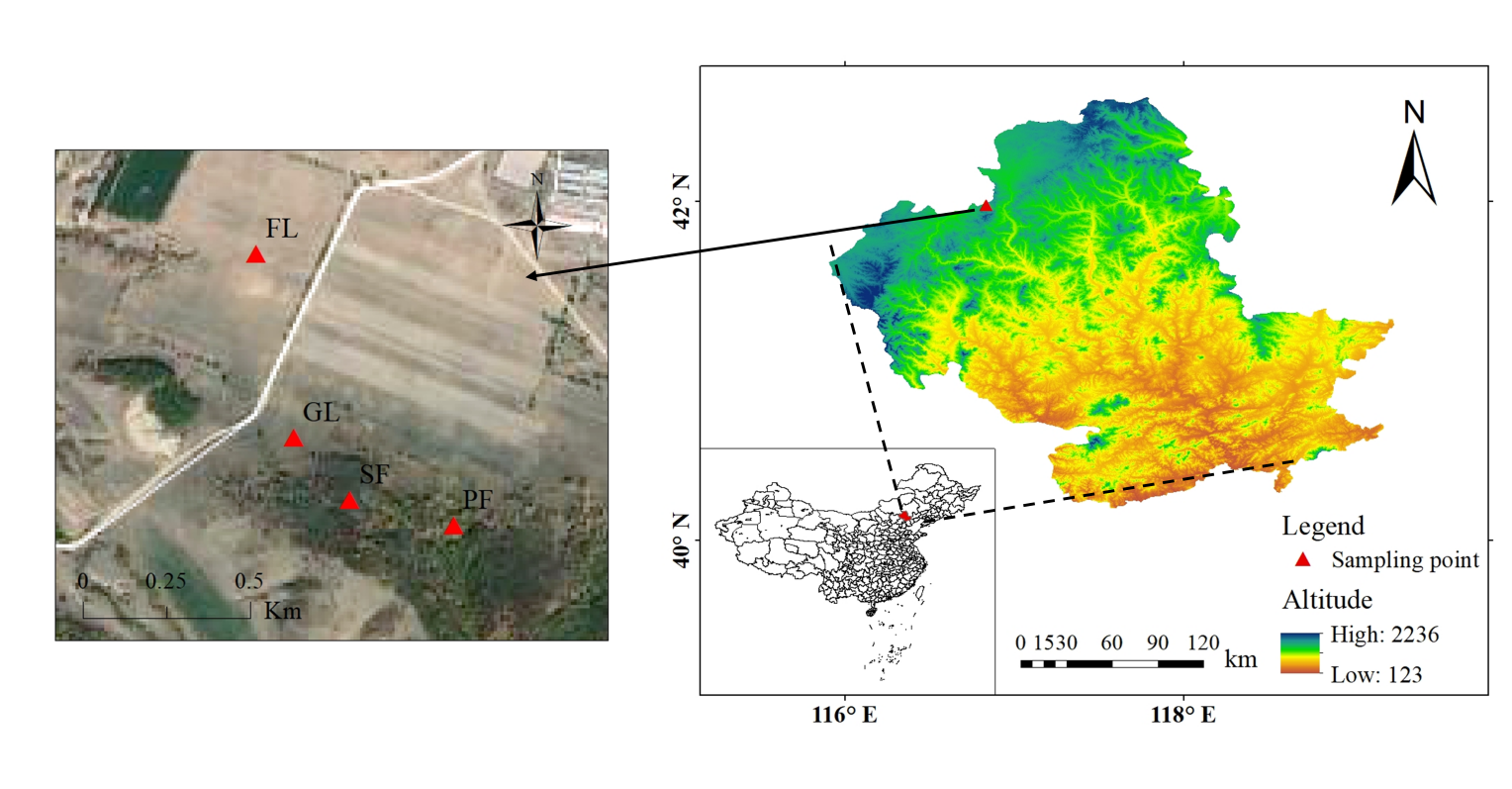


Supplementary Figure 1. The geographical location map of the study area.

Note: FL: farmland; GL: grassland; SF: secondary forest; PF: planted forest.

**Supplementary Table 1. Basic overview of the sample plot**

| Land use types | Elevation/(m) | Mean BDH/(cm) | Mean tree height/(m) | Crown density | Cover degree/% |
| --- | --- | --- | --- | --- | --- |
| Farmland | 1357 | / | / | / | 80 |
| Grassland | 1375 | / | / | / | 77 |
| Secondary forest | 1402 | 14.44 | 11.2 | 0.70 | / |
| Planted forest | 1406 | 13.75 | 10.8 | 0.53 | / |

**Supplementary 2: Specific methods for determining amino sugar**

Weigh 0.3 mg nitrogen‑equivalent soil sample into a hydrolysis flask, add 10 mL of 6 mol·L⁻¹ hydrochloric acid, and hydrolyze for 8 h. After the filtrate is dried, adjust its pH to 6.6–6.8, transfer it to a centrifuge tube, and centrifuge at 3000 r·min⁻¹ for 10 min to remove precipitates. After discarding the precipitates, add 4 mL of anhydrous methanol for dissolution, and centrifuge again at 3000 r·min⁻¹ for 10 min to desalt. Transfer the supernatant to a 5 mL derivatization vial, dry under nitrogen flow, and then freeze‑dry again. After the sample is completely dried, add derivatization reagent for derivatization. Subsequently, dissolve the product with dichloromethane, add hydrochloric acid, and vortex. After removing the upper inorganic phase, dry the remaining organic phase under high‑purity nitrogen flow, and dissolve it in 400 µL of ethyl acetate‑n‑hexane (volume ratio 1:1). Separation and detection of the product are performed using a gas chromatography‑mass spectrometer (Agilent 7890A‑5975C, USA). Record the retention times of samples and standards, compare them to identify the peaks of amino sugar derivatives, and use the pre-purified inositol added to the samples as an internal standard for quantitative analysis of amino sugars.
